# A Rare Emergency: Testicular Torsion in the Inguinal Canal

**DOI:** 10.1155/2015/320780

**Published:** 2015-01-14

**Authors:** Nevzat Can Şener, Okan Bas, Nihat Karakoyunlu, Hakan Ercil, Suleyman Yesil, Kursad Zengin, Abdurrahim Imamoglu

**Affiliations:** ^1^Department of Urology, Numune Teaching and Research Hospital, Ministry of Health, Adana, Turkey; ^2^Department of Urology, Abdurrahman Yurtarslan Onkoloji Teaching and Research Hospital, Ankara, Turkey; ^3^Department of Urology, Diskapi Yildirim Beyazit Teaching and Research Hospital, Ministry of Health, Ankara, Turkey; ^4^Department of Urology, Gazi University School of Medicine, Ankara, Turkey; ^5^Department of Urology, Bozok University School of Medicine, Yozgat, Turkey

## Abstract

*Objectives*. To report our experience and present the largest series of testicular torsion cases in the inguinal canal. *Material and Methods*. The clinical data of 13 patients with testicular torsion in the inguinal canal treated between 2005 and 2013 were reviewed. Recorded patient age, whether the testes were palpable or not, side of the affected testes, the presence of hernia, ischemia time, and operation outcomes were assessed. *Results*. Patient age ranged from 8 to 70 months (29.15 ± 20.22). Mean ischemia time was 16.5 ± 21.3 hours. Accompanying inguinal hernia was present in 92% of the cases (12/13). Four of the thirteen patients (30.8%) were treated by orchiectomy because the necrosis was present after prolonged ischemia time. Nine patients (69.2%) were treated by single session orchidopexy. *Conclusion*. Torsion of testes in the inguinal canal is a rare disease, but with rapid diagnosis, affected testes can be salvaged, but the key factor is to keep this condition in mind.

## 1. Introduction

Testicular torsion is a urologic emergency that needs immediate attention and treatment. The rotation of the spermatic cord leads to venous and arterial flow compromise, which results in ischemia in about 6–24 hours [1].

Undescended testis, or cryptorchidism, is usually present at birth. About 1/3 premature male newborns have this condition [2]. One percent of term male infants suffer from cryptorchidism [2]. It is the most common congenital anomaly affecting genitalia of newborn male infants [3].

The most common symptom of testicular torsion is scrotal pain. However, when testes are undescended, like testicular torsion in the inguinal canal, the diagnosis becomes harder for the physician. The incidence of torsion of cryptorchidism is thought to be higher than torsion of the scrotal testes [4]. However, there are limited data on testicular torsion in the inguinal canal. In this paper, we aimed to report our experience and present the largest series of testicular torsion cases in the inguinal canal.

## 2. Patients and Methods

Between 2005 and 2013, 217 patients were treated in our clinic for testicular torsion. Of these patients, 13 had testicular torsion in the inguinal canal. The clinical data of these patients were reviewed. Age at presentation, associated anomalies, and operation outcomes were studied.

Diagnosis of testicular torsion was confirmed by physical examination and scrotal Doppler ultrasound (SDU).

Recorded patient age, whether the testes were palpable or not, side of the affected testes, the presence of hernia and ischemia time, and operation outcomes were assessed.

Statistical analyses were performed using Statistical Package for Social Sciences (SPSS) 20 software for MAC (SPSS Inc., Chicago, IL, USA). All the data are presented as mean (range).

## 3. Results

Clinical presentations consisted of irritability, persistent crying, swelling, and pain in the inguinal region. Age of the patients ranged from 8 to 70 months (29.15 ± 20.22). All the testes were palpable in the inguinal region. The affected testes were 38.5% right and 61.5% left.

The mean ischemia time was 16.5 ± 21.3 hours. Accompanying inguinal hernia, which was detected intraoperatively, was present in 92% of the cases (12/13). Hernia repair was performed on these patients.

Four of the thirteen patients (30.8%) were treated by orchiectomy because the necrosis was present after prolonged ischemia time. Nine patients (69.2%) were treated by single session orchidopexy.

Patients were followed up for a mean of 23.7 months. From nine patients treated by orchidopexy, two patients suffered from testicular atrophy at a mean of 11.5 months.

Patient characteristics and operative outcomes were summarized in Table 1.

## 4. Discussion

Testicular torsion is a real urologic emergency. Sudden diagnosis and intervention should be made because it may result in the loss of the testicle. With immediate recognition and management, 40 to 60% of testicles can be salvaged [5, 6]. Davenport proposed a salvage rate of 90% with the diagnosis of testicular torsion in six hours after the incident, whereas the salvage rate decreases to less than 10% after 24 hours [1].

Cryptorchid or undescended testis is classified as abdominal, inguinal, or subinguinal. About 75% of undescended testis is classified as inguinal [7]. Cryptorchidism may cause lower sperm count, worsened sperm quality, and lower fertility rates. These conditions are worse when undescended testis is bilateral and orchidopexy is performed at an older age [8]. Gaudio et al. demonstrated the decrease of germ cell density in cryptorchidism after 1 year of age [9].

There is a significantly higher risk of testicular tumors in patients with undescended testis among normal population (1/1000–2500 to 1/100,000) [10]. Management with orchidopexy has not been proven for risk reduction but detection by self-examination is eased with orchidopexy [11].

Cryptorchid testis is more prone to torsion, compared to normal testicle. There are articles proposing 10 times higher risk of torsion in cryptorchid testicle [12, 13]. Even though it is more frequent, the literature of torsion of undescended testicle is mostly limited to case reports [14, 15]. The reports propose a rapid intervention and mostly focus on diagnostics. Erdogan et al. report a case with inguinal hernia, where sudden onset of symptoms gave rise to suspicions of testicular torsion, even with complicated presentation [16].

As important as duration, degree of cord twisting is a critical factor for the outcome of testicular torsion [17]. When twisting is >360 degrees, torsion as short as 4 hours may facilitate testicular atrophy. If the torsion is not complete (<360 degrees), testicles with torsion within 24 hours may be salvaged [18]. With the presence of complete torsion (360 degrees), severe testicular atrophy was reported. However, with incomplete torsion (<360 degrees), atrophy was not observed even within 12 hours [18].

For differential diagnosis, the most important factor is physician's experience. One must always keep the undescended testicle in mind when an inguinal mass and tenderness are present. However, testicular torsion may not always be the case. Orchitis of undescended testicle, incarcerated inguinal hernia, and tumor of the undescended testicle should be kept in mind. Scrotal Doppler ultrasonography usually helps supporting the diagnosis [19–21].

In this report, 13 patients with testicular torsion in the inguinal canal and torsion were treated. To our knowledge, this is the largest series concerning testicular torsion in the inguinal canal. The mean patient age was 29.15 ± 20.21 months. The ages of patients were higher than the age where they should be treated. This is because the majority of our patients were from the countryside and healthcare could not be provided optimally for those who live in countryside in our country. All testes were in the inguinal canal and were palpable. Because the physician can palpate the affected testicle, testicular torsion in the inguinal canal is easier to diagnose among other cryptorchid cases. For example, intra-abdominal testis cannot be palpated like other testicular problems but should be kept in mind with the presence of abdominal pain and a missing testicle. It is reported to be a rare cause of acute abdomen [13]. Inguinal hernia is mostly associated with testicular torsion in the inguinal canal [22], and, in our report, this ratio is 92%, similar to those in literature. The mean ischemia time was 16.54 ± 21.25 hours. However, we had one case with a history of 84 hours. If this case is neglected, the average is 10.07 ± 7.06 hours, which is compatible with the literature. We could be able to salvage 69.2% of the affected testes. In 30.8% of the cases, affected testes had to be removed. Torsion angle of the testicle is an important factor for treatment outcomes. One patient in our series with a complete torsion was successfully operated on in 4 hours with the testicle salvaged. However, in another patient with an onset of 11 hours with complete torsion, the operation resulted in orchiectomy.

Even though this study lacks long-term follow-up and has a retrospective method with a very small number of patients, we believe it may yield important information because of the lacking data in current literature and aid physicians to diagnose this infrequent disease.

## 5. Conclusion

Torsion of cryptorchidism is a rare disease, but, with rapid diagnosis, affected testes can be salvaged. Physical examination is the key to diagnosis. Physicians should suspect testicular torsion in the inguinal canal with a missing testis and a tender mass in the ipsilateral inguinal region.

## Figures and Tables

**Table 1:** Characteristics and outcomes of patients.

Patient	Age (months)	Side	Symptom duration (hours)	Necrosis?	Torsion degree	Outcome	Follow-up
1	16	Right	7	No	270	Orchidopexy	
2	22	Left	8	No	180	Orchidopexy	
3	15	Left	11	Yes	360	Orchidectomy	—
4	24	Left	26	Yes	360	Orchidectomy	—
5	18	Left	22	Yes	180	Orchidectomy	—
6	70	Left	84	Yes	270	Orchidectomy	—
7	16	Left	8	No	270	Orchidopexy	
8	8	Right	4	No	270	Orchidopexy	
9	23	Right	11	No	270	Orchidopexy	Atrophy
10	22	Right	10	No	180	Orchidopexy	
11	56	Left	4	No	360	Orchidopexy	
12	64	Right	8	No	270	Orchidopexy	
13	25	Left	12	No	180	Orchidopexy	Atrophy
